# Management of Cardiac Involvement in NeuroMuscular Diseases: Review

**DOI:** 10.2174/1874192400802010093

**Published:** 2008-10-22

**Authors:** Rachida Bouhouch, Tarik Elhouari, Latifa Oukerraj, Ibtissam Fellat, Jamila Zarzur, Rajaa Bennani, Mhamed Arharbi

**Affiliations:** Service de Cardiologie B, CHU Ibn Sina Rabat, MAROC

**Keywords:** Muscular dystrophy, cardiomyopathy, sudden cardiac death.

## Abstract

Neuromuscular Diseases are a heterogeneous molecular, clinical and prognosis group. Progress has been achieved in the understanding and classification of these diseases.

Cardiac involvement in neuromuscular diseases namely conduction disorders, ventricular dilatation and dilated cardiomyopathy with its impact on prognosis, is often dissociated from the peripheral myopathy. Therefore, close surveillance is mandatory in the affected patients. In this context, preventive therapy (beta-blockers and angiotensin converting enzyme inhibitors) has been recently recommended in the most common Neuromuscular Diseases, Duchenne Muscular Dystrophy and Myotonic Dystrophy.

## INTRODUCTION

During last years, progress has been achieved in the understanding and classification of neuromuscular diseases.

Cardiac involvement (Table **1**) in the neuromuscular diseases namely conduction disorders, ventricular dilatation and dilated cardiomyopathy with its impact on prognosis, is often dissociated from the peripheral myopathy. Therefore, close surveillance is mandatory in affected patients. In this context, preventive therapy has been recently recommended in the most common forms the Duchenne Muscular Dystrophy and the Myotonic Dystrophy.

### Duchenne (DMD) and Becker (BMD) Muscular Dystrophy

In the DMD, prognosis depends on the ventilatory assistance (respiratory muscle dysfunction is the main reason for death, survival thus depend on the ventilatory support). In BMD, prognosis rather depends on the existence of a cardiomyopathy.

Both diseases are X-linked recessive disorders due to the Dystrophine gene abnormality with almost absence of Dystrophine in DMD and reduced Dystrophine in BMD [1-2].

### Epidemiology and Clinical Presentation (Table 2)

DMD is the most common inherited neuromuscular disorder with an incidence of 30/100000 live male births. A cardiomyopathy is always present but is masked by severe muscle weakness. The electrocardiogram (ECG) is often abnormal. Death occurs by the age of 20 years old in DMD versus 40-50 years old in BMD [3].

### Cardiac Management (Table 3)

Angiotensin Converting Enzyme Inhibitors (ACEI) have been shown to be effective in preventing left ventricular (LV) remodeling in ischemic cardiomyopathy and reducing LV hypertrophy and fibrosis which contribute to ventricular arrhythmias and sudden cardiac death (SCD) [4]. Recently, ACEI have been studied in patients with muscular dystrophies. Early initiation of treatment with perindopril was associated with lower mortality in patients with DMD with normal LV ejection fraction at study entry [5]. Likewise, others [6] found that a beta-blocker (BB), in addition to ACEI improves LV systolic function in patients with muscular dystrophy.

In the light of these positive results, many authors [5-6] recommend initiation of ACEI and/or BB early after diagnosis of the muscular dystrophy especially in DMD.

Some studies suggested that corticosteroids might have a beneficial effect in DMD [7].

Males with DMD must be followed-up more closely than female with DMD or those with BMD [7].

### Myotonic Dystrophies

The most common form is Myotonic Dystrophy 1(MD1) also known as the Steinert disease with an incidence of 1/8000 births [1-10].

It is an autosomal dominant disorder due to mutational expansion of a repetitive trinucleotide sequence (CTG) in the 3'-untranslated region of the DMPK (myotonic dystrophy protein kinase gene) on chromosome 19q13.3. SCD is the major risk in affected patients and may occur at a very young age. [8-9]

### Epidemiology and Clinical Presentation (Table 4)

MD1 is the most common form of MD in adults between 20 and 25 years old [1-10].

The mean age survival is 53 years old. Cardiovascular Events (LV dysfunction, ischemic heart disease, pulmonary emboli or SCD) represents 30% of all cause mortality [10].

Moreover, cardiac involvement may be the first expression of the disease [10].

#### Conduction Disorders

First degree AV bloc is seen in up to 40% cases. Bundle branch blocs, a long QT, ST-T modifications and axis deviations are other possible findings [10-11].

Because progression of these conduction disorders is unpredictable, in addition to the high prevalence of infra-His defects (conduction impairment below the His bundle), an electrophysiology study (EPS) should always be performed.

Late potentials (LP), rather than a risk indicator for ventricular arrhythmias, seems to correlate with a delayed activation along the His-Purkinje system [10-12]. LP can thus be used for selection of candidates for an EPS (Table **5**).

Asymptomatic sinus bradycardia has been reported in pediatric series [13].

#### Tachycardia

- Supraventricular tachycardias are very common with up to 25% patients presenting atrial fibrillation or and/or atrial flutter [10-12].

- Ventricular tachycardias (VT) are frequent, thus any symptomatic patient should undergo an EPS (Table **5**). 50% of patients who had a pace-maker (PM) for AV bloc, develop VT and are at risk of SCD. Different mechanisms are described for the VT, a particular one is the bundle branch rerentry (BBR) VT since it can be cured by radiofrequency ablation [10,12,14].

#### Cardiomyopathy and other Disorders

Patients present symptoms of heart failure in only 1.8% cases because they are usually limited in their daily activity. In fact, cardiac imaging can unmask 14% of asymptomatic LV dysfunction [10-12].

### Cardiac Management [10,12,13]

While waiting for several ongoing studies that will evaluate arrhythmias and cardiac manifestations of DM1, suggestions for management of cardiovascular complications in DM1 patients are as follows:

An ECG every 6 to 12 months is advised in asymptomatic patients with a normal baseline ECG.

EPS is indicated as previously mentioned in Table **5**. 

According to recent guidelines [15], PM implantation is a class1 indication in the presence of a third degree and advanced second degree AV block at any anatomic level with or without symptoms. If a VT at EPS is only a BBRVT, radiofrequency ablation of 1 BB (usually the right BB) is probably curative and the patient may not need an implantable cardioverter defibrillator (ICD) if he/she does not meet the classical criteria for ICD placement and if there is no other inducible VT with a mechanism other than BBR.

Echocardiography is recommended in symptomatic patients and in those patients with conduction disturbances or arrhythmia.

### Emery-Dreifuss Dystrophies (EDMD)

#### Epidemiology and Clinical Presentation

Two forms are described; EDMD2 is the autosomal dominant type caused by mutations of the LMNA gene at 1q21 (which encodes lamins A and C); EDMD1 is the X-linked form caused by mutations of the STA gene at Xq28 (which encodes the nuclear membrane protein emerin) [1-2].

ECG is abnormal by age 20 to 30 years old with sinoatrial or auriculo-ventricular conduction disorders, atrial arrhythmias, ventricular arrhythmias (particularly in EDMD2) [13]. Cardiomyopathy (CMP) represents 2% of CMP in children and may develop earlier in the EDMD2 [13].**

Survival is 35% at the age of 45 years old with a high risk of SCD [1-2-11].

#### Cardiac Management

Family members of the EDMD, especially EDMD2, must be screened after the age of 10 years old, even in the absence of symptoms [13].**

It is advised to perform during follow-up, an ECG, a Holter ECG and an Echocardiography once a year. An exercise test may be helpful in children and an EPS will be indicated as for the MD1.

Anticoagulation therapy must be started in the setting of atrial arrhythmia or atrial standstill [13]. An ICD is indicated in the EDMD2 associated with dilated cardiomyopathy especially in the adulthood [1-13].

### Limb-girdle Muscular Dystrophies (LGMD)

Several of these disorders are associated with clinically significant cardiac involvements [1,2,11,13].

#### LGMD Type 1B

In this autosomal dominant form, conduction disorders progression resembles EDMD2 that can be relevant of a PM or an ICD [1-13].

#### Other LGMD

##### Type 2C, 2D, 2E, 2F LGMD

They are sarcoglycanopathies due to defects on gamma, alpha, beta and delta sarcoglycans.

Cardiomyopathy is possible except in alpha-sarcoglycanopathy.

Fanin reported cardiomyopathy in 10 patients with LGMD type 2E from 6 different families [11]; atrial fibrillation was found in 2 out of these 10 patients.

Though guidelines are lacking in these dystrophies, it is advised to repeat ECG/Holter and echocardiography every 2 to 5 years. Empirical therapy by ACEI and /or BB is used in the setting of LV dysfunction [13].

##### Type 21 LGMD,

is an alpha-sarcoglycanopathy that associates a dilated cardiomyopathy in 50% cases [13].

##### Calpainopathies(LGMD 2A) and Dysferlinopathies,

have a Becker phenotype except for the cardiac involvement [13].

### The Facioscapulohumeral Muscular Dystrophies (FSHMD)

Described by Landouzy and Dejerine, it is an autosomal dominant disease due to a deletion on the 4q35 chromosome. Arrhythmias are rare in this muscular dystrophy [1-11].

#### Mitochondrial Myopathies

It is a heterogeneous group due to mutations in mitochondrial DNA. Two phenotypes are described: [1,11,13]

*1. The Kearns-Sayre syndrome oculo-pharyngal myopathy*, with chronic progressive external ophtalmoplegia. Infra-His conduction disorders are progressive [1-11]. Therefore, an EPS is performed in symptomatic patients and in presence of ECG abnormalities. Cardiac pacing improves survival of patients with a prolonged infra-His conduction interval at the EPS. Patients with ventricular arrhythmias may require an ICD[13-15].

*2. The Melas syndrome and Leber disease* ,may associate a supraventricular tachycardia due to an accessory pathway [1-11].

## CONCLUSIONS

Advances in the understanding of the incidence, the type, the pathophysiology and molecular biology of the various peripheral myopathies and their cardiac complications had allowed recommendations for the cardiological follow-up of the patients especially in the commonest diseases (Steinert's disease, Duchenne and Becker muscular dystrophies and EDMD). Little is known regarding the management of cardiac complications in children with the same diseases; prospective studies in this age group of patients are therefore mandatory. This may lead to early screening in affected families and adjustment of guidelines for device implantation in adults for this young population.

## Figures and Tables

**Table 1.** Risk of Cardiac Complications in Neuromuscular Diseases

Neuromuscular Diseases	Conduction Disorders / Ventricular Arrhythmias	Cardiomyopathy
DMD & BMD	+ / +	+++ & ++
Steinert Disease	+++ / +++	+
EDMD A/C EDMD X-linked	+++ / +++ +++ / ++	++ ++
LGMD 1B LGMD 2C 2F	+++ / +++ + / +	++ ++
Kearns-sayre syndrome	+++ / ++	+

DMD: Duchenne muscular dystrophy; BMD: Becker muscular dystrophy; LGMD: Limb girdle muscular dystrophy; EDMD: Emery dreifuss muscular dystrophy; +: Low risk; ++:  moderate risk; +++: High risk.

**Table 2.** Epidemiology and Clinical Presentation of DMD and BMD

	BMD	DMD
Incidence	3/100.000 live male births	30/100.000 live male births
Cardiac involvement	CMP** 50%	CMP* 25% after 6 y/o
ECG / Holter ECG	Abnormal 75%	Abnormal 90% Sinus tachycardia 26%
Progression***	More benign(survival → 40-50 y/o)	Rapid (symptoms > 5 y/o, death ~ 20 y/o)

* Masked by the severity of the peripheral myopathy.

** The right ventricular may be the first to be involved.

*** MRI (Magnetic Resonance Imaging) could be used to monitor disease progression and possibly response to therapy. CMP, Cardiomyopathy; y/o, years old.

**Table 3.** Cardiac Management of DMD and BMD

	BMD	DMD
Corticosteroids	Unknown	May be beneficial
ACEI ≥ 9 y/o Diuretics, BB and anti-arrhythmics if CHF	Unknown	Would be beneficial
Follow-up	♂, ECG+TTE every 5 years ♀asymptomatic, ECG+TTE after 16 y/o	♂, ECG+TTE every 2 years →10 y/o then once a year ♀asymptomatic, ECG+TTE every 5 years after 16 y/o

ACEI, Angiotensin converting enzyme inhibitors; BB, BetaBlockers; CHF, Congestive heart failure; TTE, TransThoracic echocardiography.

**Table 4.** Epidemiology and Clinical Presentation of Myotonic Dystrophies

	MD2 or PROMM	MD1 Steinert
Genetic alterations	Repetitive CCTG Chr 3	Autosomal dominant repetitive CTG Chr 19q13.3
Neurological disorder	Proximal	Distal
SCD Risk	Less common & late	Common and early

PROMM, PROxymal myotonic myopathy; SCD, Sudden cardiac death.

**Table 5.** Indications for EPS in DM1 Patients

Family History	Sudden death, Ventricular Fibrillation/Sustained VT, Pacemaker or cardioverter defibrillator implant
Symptoms	Palpitations, Syncope, Dizziness
Conduction disorders at ECG/HolterECG/Signal averaged ECG	First degree AV block Second or third degree AV block QRS > 120 ms LBBB, RBBB+LAFB Positive late potentials
Sinus node dysfunction	Sinus pause > 3 seconds Sinus bradycardia < 40 / min
Supra-Ventricular arrhythmias	Atrial tachycardia AF/A FLT
Ventricular arrhythmias	Frequent ventricular premature beats Non-sustained or Sustained VT

AV, atrioventricular; AF, atrial fibrillation; A FLT, atrial flutter; LAFH, left anterior fascicular hemiblock; LBBB, left bundle branch block; RBBB, right bundle branch block; VT, ventricular tachycardia.
